# Efficacy of Heart Revival, an Ayurvedic formulation, in hypertension and related risks – An exploratory single arm open label trial

**DOI:** 10.1016/j.jaim.2024.100975

**Published:** 2024-07-23

**Authors:** Srikanta Pandit, Tuhin Kanti Biswas, Sayantan Bera, Sumana Saha, Utpalenedu Jana, Tapas Kumar Sur

**Affiliations:** a Department of Kayachikitsha, J.B. Roy State Ayurvedic Medical College & Hospital, Kolkata, West Bengal, 700 004, India; b Multidisciplinary Research Unit (ICMR), R.G. Kar Medical College & Hospital, Kolkata, West Bengal, 700 004, India

## 1 Introduction

In 2016, WHO reported nearly 17.9 million of all global demises were attributable to cardiovascular diseases (CVDs) [1]. CVDs are a group of dysfunctions of heart and blood vessels including, coronary heart disease, cerebrovascular disease, peripheral arterial disease, rheumatic heart disease, congenital heart disease and deep vein thrombosis and pulmonary embolism. The common risk factors of CVDs are diabetes, hypertension, hyperlipidemia, physical inactivity, obesity and harmful use of tobacco and alcohol [2]. WHO estimated due to CVDs, India loses $237 billion for spending on medical care over a 10-year period (2005–2015) [3].

Hypertension has been consistently correlated with increased probability of rising CVDs [4]. The epidemiological studies and other experimental evidence supported that hypertension influences to accelerate atherosclerosis through a shared synergistic mechanism involving inflammation and oxidative stress in the arterial wall [5]. However, several strategies are taken to reduce blood pressure to delay or prevent atherosclerotic lesion [6]. Moreover, the use of anti-hypertensive drugs showed an association with late CVDs. β-blockers improve the endothelium dependent vasodilatation and also decrease peripheral vascular resistance [7]. Hyperlipidemia or more specifically hypercholesterolemia is another key risk factor of CVDs. Atorvastatin, an inhibitor of HMG-CoA reductases provide a very effective means of reducing LDL-cholesterol concentrations in hyperlipidemia/hypercholesterolemia [8]. Although long term risks of the statin are not fully known, perhaps recent controversy whether statin cause type 2 diabetes [9]. Besides that, high triglyceride-rich lipoproteins or remnant cholesterol and electrolyte abnormalities in cardiovascular emergencies are widely studied all over the world. Actually, sodium balance is essential to maintain plasma osmolality and blood pressure [[10], [11], [12]]. Nevertheless, a number of scientific committees recommended for the use of balanced cardio protective diet as preventive measure of CVD [13].

From ancient times, Ayurveda, traditional system of India medicine described the factors related to the origin, formation and structural relations of Heart (*Hridaya*) and types of various heart diseases (*Hridroga*). In these texts, many compounds and formulations are found for the management of heart diseases [14,15]. Medicinal plants possesses anti-platelet, antihypertensive, hypotensive, hypolipidemic, anti-inflammatory and hypoglycemic actions. Based on Ayurvedic concepts and literature, a new formulation Heart Revival (M/s Health Reactive, Rajasthan, India) has developed. The major ingredients of Heart Revival are *Arjuna* (*Terminalia arjuna*)*, Ashwagandha* (*Withania somnifera*)*, Lahsun* (*Allium sativum*), *Anar (Punica granatum), Giloy* (*Tinospora cordifolia*)*, Amla* (*Phyllanthus emblica*)*,* Guggulu (*Commiphora wightii*) and *Dasmula (a group of ten ayurvedic medicinal plants)*. Experimental studies reported it has significant cardio-protection, anti-platelet, blood lipid lowering and antioxidant properties [16,17]. Each 5 ml of Heart Revival contains purified water extract of Arjuna (30 mg), Ashwagandha (15 mg), Lahsun (15 mg), Anar (15 mg), Giloy (15 mg), Amla (10 mg), Guggulu (25 mg) and Dasmula (10 mg) in honey*. T. arjuna* has reported for reducing blood pressure, serum cholesterol and anginal frequency [18]. *A. sativum* has known for antihypertensive, hypocholesterolemic and anti-thrombotic actions [19]. *E. officinalis* possesses cardiac specific antioxidants and exhibited myocardial and endothelial function [20]. Polyphenolic compounds of P. granatum seeds exhibited potential action on ischemic heart disease [21]. *T. cordifolia* have reported for anticoagulant, platelet antiaggregatory and hypolipidaemic properties [22]. Besides that, in combination the formulation acts as rejuvenation of entire body and therefore, recommends in a single dose at night.

Therefore, the primary objective of the present study was to find out the therapeutic role of Heart Revival suspension on adult participants who were suffering in mild to moderate CVDs, particularly hypertension, hypercholesterolemia, arrhythmia, electrolyte disturbance, heart function, and blood circulation. The secondary objective of the study was to measure the safe and long term use of medicine.

## 2 Materials and methods

### 2.1 Study design

This study was single arm open labeled clinical trial conducted at the Research Unit, Department of *Kayachikitsha*, J.B. Roy State Ayurvedic Medical College & Hospital, Kolkata, between the time periods of 2020–2021. The study was conducted in accordance with provisions of the Declaration of Helsinki, the Guidelines of Good Clinical Practice and applicable to ethical guidelines for biomedical and health research involving human participants by Indian Council of Medical Research, Govt. of India, 2018 [23]. The trial sponsor, M/s Health Reactive, Jaipur, Rajasthan, India provided the logistic support. The data was analysed and interpreted by the investigators in collaboration with all authors. All the authors contributed to the drafting, review and revision of the manuscript.

### 2.2 Ethical approval

The trial protocol was approved by the Institutional Ethics Committee (JBR/RU/CT-14/2019, dt. 27/5/2019) and Clinical Trials Registry of India (Regn No.: CTRI/2020/01/022974 dt. 27/1/2020).

### 2.3 Study participants

Adult participants of either sex, who were complained for any history of cardiac problems, were registered for the trial. The history of hypertension, hypercholesterolemia, electrolyte disturbance, arrhythmia, heart function and blood circulation were given preferences.

### 2.4 Screening methods

Each participant was included in the study after their written consent in prescribed format. After receiving the written consent, the patient was registered for screened for study related examinations. The clinical parameters (BMI, BP, HR), chest x-ray, ECG and Echo-cardiogram (2-D & M-Mode) were checked. Fasting blood (10 ml) was withdrawn to estimated hematological and biochemical parameters (CBC, glucose, lipid profile, LFT, renal function, electrolytes).

### 2.5 Selection criteria

After screening, the eligibility criteria were checked. The inclusion criteria were: either sex, age between 18 and 60 years, baseline hypertension BP < 130/89 mm of Hg, hyperlipidemia CHO<150 mg/dl or LDL: CHO >3, not receiving any hypertensive or lipid lowering medicine and willing to give writing consent [6,8]. The exclusion criteria were: aged below 18 years and above 60 years, third degree heart block, chronic antipsychotic drug history, suffering from major cardiac ailments, uncontrolled diabetes (blood glucose, PP > 400 mg/dl), chronic kidney disease, pregnant and lactating mother.

### 2.6 Enrollment and intervention

Selected participants were enrolled for the treatment procedure. All enrolled participants were advice to receive 5 ml of Heart Revival once daily, orally at the time of bed (preferably night) for 60 consecutive days and also requested for not to use any antihypertensive medicine or any other medicines without consult with the investigator's team. The therapeutic effective dose has been selected on the basis of previous experimental data and dose conversion rule [16,17]. After 30 days, all participants were informed to follow up visit. Finally, all participants were thoroughly (similar to screening visit) examined at day 60. In this trial period, all participants were advice not to take any hypertensive or lipid lowering medicines.

### 2.7 Criteria of assessments

#### 2.7.1 Primary outcome measure

Peripheral blood pressure (systolic and diastolic), ECG, Echo-Cardiogram (2-D & M-Mode), Chest X-ray (PA view) and fasting blood examination (glucose, total Cholesterol, HDL-Cholesterol, LDL, VLDL, triglyceride, Na^+^, K^+^, Cl^−^, HCO^−^_3_). All assessments were evaluated at baseline and at day 60 of interventions. The primary end points of the trial were hypertensive and hyperlipidemic response (lowering blood pressure and blood lipids) that was sustained at two scheduled assessments at weeks 4 and 8 during the fixed-dose period.

#### 2.7.2 Secondary outcome measure

For safety evaluation of study intervention, complete blood count (Hb, RBC, PCV, MCV, MCH, MCHC, WBC, Platelet, DC), Liver Function Tests (Protein, Albumin, Globulin, SGOT, SGPT, Alkaline phosphatase, Total bilirubin, Conjugated bilirubin, Un-conjugated bilirubin) and Renal Function Tests (Urea, Creatinine) were conducted at baseline and at day 60 of interventions.

### 2.8 Statistical methods

Outcome measures of efficacy were performed in the full set of all participants who had continued treatment until the end of the study. Summary statistics was comprised of group means and standard deviation (SD). The primary end point variables especially, hypertension response was analysed within and between the male and the female participants.

The primary and secondary categorical data between the baseline and post treatment (at 4 weeks and 8 weeks, whatever applicable) were compared with the paired *t*-test using statistical software (SPSS version 20; IBM Corporation, Chicago, Illinois, USA). Safety outcomes were summarized descriptively and separately. The level of statistical significant was considered as p < 0.05.

## 3 Results

### 3.1 Primary outcome measure

The baseline demographic data is presented in Table 1. Out of 45 complete participants 14 were male. The age, socioeconomic status and other baseline variables are provided as Table 1. The medical history revealed other than hypertension, hypercholesterolemia was noted in 39, diabetes in 11 and mild cardiac complications RBBB, ST depression, sinus rhythm etc. in 8 participants. None of them use any hypertensive medicine.

**Table 1:** Baseline demographic data

Distribution of participants	Category	Frequency
Enrolment	Screened	48
	Screen failure	2
	Drop out	1
	Trial Completed (N)	45
Gender	Male	14
Female	Female	31
Age	18–40 years	15
	41–60 years	30
Socio economic status	Middle class	8
	Lower class	37
Diet	Vegetarian	1
	Mixed	44
Salt intake in diet	Normal (<5 g/day)	42
	Moderate (5–9 g/day)	3
Smoking	Non Smoker	34
	Smoker/tobacco user	11
Physical activity	Normal (less than 1000 calories/day)	41
	Moderate (1000–3000 calories/day)	4
Medical History	Hypertension	45
	Diabetes	11
	Hypercholesterolemia	39
	ECG: RBBB, ST depression, Sinus rhythm	8
	Echo: Normal	27
Clinical complains	Fatigue	34
	Chest Pain/discomfort/breathing trouble	31
	Irritability	25
	Palpitation	21
	Headache	20

The data of physical assessments of trial participants is given Table 2. The test medicine Heart Revival was given in all participants for 8-week. Heart Revival did not significantly change BMI, pulse rate and respiration rate. However, both systolic and diastolic BP was consistently and significantly (p < 0.001) lowered after the drug treatment in hypertensive participants.

**Table 2:** Physical assessments of the trial participants.

Category		Baseline (Mean ± SD)	4 Week (Mean ± SD)	p-value	8 Week (Mean ± SD)	p-value
BMI	Male	25.26 ± 4.05	24.80 ± 3.71	NS	24.66 ± 3.72	NS
	Female	25.66 ± 4.06	25.48 ± 3.97	NS	25.38 ± 3.96	NS
Pulse rate (min)	Male	76.50 ± 9.23	75.93 ± 8.41	NS	74.35 ± 8.06	NS
	Female	77.87 ± 8.90	77.03 ± 8.33	NS	76.83 ± 7.99	NS
BP–Systole (mm Hg)	Male	148.78 ± 8.78	130.71 ± 4.58	<0.001^(a)^	124.57 ± 3.83	<0.001^(a)^
	Female	146.77 ± 7.50	130.58 ± 4.55	<0.001^(a)^	124.54 ± 4.64	<0.001^(a)^
BP-Diastole (mm Hg)	Male	98.78 ± 3.05	86.92 ± 5.85	<0.01^(a)^	82.57 ± 3.63	<0.001^(a)^
	Female	97.19 ± 6.01	84.96 ± 4.22	<0.001^(a)^	82.29 ± 3.49	<0.001^(a)^
Respiration (min)	Male	21.14 ± 2.17	20.78 ± 1.88	NS	20.57 ± 1.94	NS
	Female	21.32 ± 2.21	20.67 ± 1.68	NS	20.41 ± 1.96	NS

All data compared to baseline of within groups (a) and between groups i.e., male vs. female (b); statistically analysed by paired *t*-test; NS = non significant.

The data of blood profile is represented in Table 3. Treatment with Heart Revival significantly lowered the total lipid profile, particularly total cholesterol (p < 0.001), LDL-cholesterol (p < 0.01), VLDL-cholesterol (p < 0.01), CHO/HDL (p < 0.05) and triglycerides (p < 0.01) in blood. Although it did not alter glucose, HDL-cholesterol and minerals in blood like sodium, potassium, chloride and bicarbonate.

**Table 3:** Blood parameters of the trial participants.

	Baseline (Mean ± SD)	8 Week (Mean ± SD)	Reference range	p-value
Glucose (F)	97.21 ± 14.58	96.02 ± 13.91	60-100 (mg/dl)	NS
Total Cholesterol	227.51 ± 21.20	179.76 ± 36.02	<200 (mg/dl)	<0.001
HDL-Cholesterol	45.48 ± 9.94	46.06 ± 9.79	40-70 (mg/dl)	NS
LDL	142.15 ± 31.82	109.82 ± 32.91	<100 (mg/dl)	<0.01
VLDL	55.28 ± 8.08	24.15 ± 7.50	0-30 (mg/dl)	<0.001
CHO/HDL	5.17 ± 1.05	4.04 ± 1.10	3.3–4.4	<0.05
LDL/HDL	3.42 ± 1.43	0.78 ± 0.13	0.5–3	<0.001
Triglycerides	140.86 ± 66.42	128.73 ± 60.51	<150 (mg/dl)	<0.01
Sodium	139.68 ± 2.33	138.59 ± 2.88	136-146 (mmol/L)	NS
Potassium	4.16 ± 0.32	4.23 ± 0.35	3.5–5.1 (mmol/L)	NS
Chloride	104.06 ± 2.35	104.05 ± 2.40	101-109 (mmol/L)	NS
Bicarbonate	24.87 ± 2.48	23.91 ± 2.45	21-31 (mmol/L)	NS

N = 45; All data compared to baseline; statistically analysed by paired *t*-test; NS = non significant.

ECG and Echocardiogram revealed that 8-week treatment with Heart Revival improved the conditions of RBBB or Right bundle branch block (in 4 patients out of 5), ST depression (4 out of 4), sinus rhythm and mild complications of Trival TR and MR (each 2 out of 3) and diastolic dysfunction (2 out of 2).

### 3.2 Secondary outcome measure

The data of hematological and biochemical safety profile are presented in Table 4. The 8-week regular treatment with Heart Revival in mild to moderate hypertensive and hypercholesterolemic participants at the dose of 5 ml/day did not altered blood morphological, hematological and metabolic functions. Moreover, Heart Revival did not interfere in QT interval of ECG.

**Table 4:** Haematological and biochemical safety profile of the trial participants.

	Baseline (Mean ± SD)	8 Week (Mean ± SD)	Reference range	p-value
Haemoglobin	12.72 ± 2.03	12.99 ± 1.84	12-15 (mg/dl)	NS
PCV	39.4 ± 5.77	41.07 ± 5.79	36-46 (%)	NS
MCV	88.12 ± 5.88	88.63 ± 6.67	83-101 (FL)	NS
MCH	29.17 ± 2.84	29.64 ± 2.88	27-32 (pg)	NS
MCHC	32.55 ± 0.94	32.41 ± 1.35	31.5–34.5 (g/dl)	NS
RBC	4.33 ± 0.52	4.42 ± 0.48	3.8–4.8 (million/cumm)	NS
WBC	6997.78 ± 1542.80	7146.66 ± 1565.47	4000-11000 (cumm)	NS
Platelet	275911 ± 50608.04	281711 ± 37947.70	150000-410000(cumm)	NS
Neutrophil	64.51 ± 6.51	65.84 ± 6.71	40-80 (%)	NS
Lymphocyte	28.93 ± 6.46	28.68 ± 6.67	20-40 (%)	NS
Eosinophil	1.80 ± 0.58	1.77 ± 0.51	1-6 (%)	NS
Monocyte	4.71 ± 1.90	4.11 ± 1.73	2-10 (%)	NS
Basophil	0.00 ± 0.00	0.00 ± 0.00	0-2 (%)	NS
Total Protein	7.38 ± 0.40	7.32 ± 0.43	6.6–8.3 (g/dl)	NS
Albumin	4.28 ± 0.26	4.24 ± 0.30	3.5–5.2 (g/dl)	NS
Globulin	3.10 ± 0.36	3.13 ± 0.40	2–3.5 (g/dl)	NS
A:G	1.40 ± 0.18	1.35 ± 0.22	1–2	NS
SGOT	27.59 ± 9.10	25.61 ± 5.40	0-35 (U/L)	NS
SGPT	25.89 ± 10.58	24.26 ± 8.82	0-35 (U/L)	NS
Alkaline Phosphatase	92.50 ± 24.46	89.41 ± 23.18	30-120 (U/L)	NS
Total bilirubin	0.76 ± 0.25	0.71 ± 0.23	0.3–1.2 (mg/dl)	NS
Conjugated Bilirubin	0.21 ± 0.04	0.23 ± 0.06	0–0.2 (mg/dl)	NS
Un conjugated Bilirubin	0.54 ± 0.23	0.49 ± 0.21	0.3–1.0 (mg/dl)	NS
Urea	18.41 ± 5.24	19.08 ± 5.91	17-43 (mg/dl)	NS
Creatinine	0.86 ± 0.17	0.76 ± 0.16	0.8–1.4 (mg/dl)	NS

N = 45; All data compared to baseline; statistically analysed by paired *t*-test; NS = non significant.

## 4 Discussion

Indian has also been experiencing an increase in the prevalence of hypertension. A cross-sectional, population-based study on a large nationally representative sample of 1.3 million individuals carried out between 2012 and 2014 revealed that the crude prevalence of hypertension in India was 25.3% [24]. It was noted even younger age groups with approximately 10% individuals aged 18–25 years [25]. Unfortunately, a large proportion of individuals are unaware of their hypertension status in this country [26].

In this study, the average age of participants was 45.5 years and BMI was 25.53. Physical inactivity in everyday lifestyle is one of the key issues for risk of CVDs. Besides physical inactivity, detrimental use of alcohol and tobacco and intake of excess salt in diet are the important concern of CVDs [27].

According to American Heart Association and American Stroke Association, in hypertensive stage I, systolic BP is 130–139 mm of Hg and diastolic BP is 80–89 mm of Hg [28,29]. Hence, mild to moderate hypertensive participants with other clinical symptoms of CVDs, like fatigue, chest pain, chest discomfort, palpitation, irritability and headache were selected for this study. Treatment with Heart Revival to all participants in a fixed dose regimen for 8 consecutive weeks significantly lowered the peripheral BP towards normal. This present observation clearly point out that the test medicine be able to prevent and control hypertension in the early stage.

Elevated levels of blood lipids are predictable risks for CVDs. Centers for Disease Control (CDC) reported hyperlipidemia is the second only to hypertension in the list of ten most deadly chronic disease conditions [8,29]. In the present study, 8 week treatment with Heart Revival significantly lowered the total cholesterol (21%) and LDL cholesterol (22.7%), ratio of CHO/HDL (21.85) and triglycerides (8.61%) in blood. Furthermore, hyperglycemia usually associated with obesity and dyslipidemia and is believed to be the possible risk for CVDs. It is evident that good glycemic control declines the risk for micro-vascular complications during diabetes [30]. In this study, the average blood sugar level was considered to be within the normal limit, although there were eleven (24.4%) diabetic participants. Heart Revival treatment did not significantly altered blood sugar level in the diabetic population.

It has been reported that reduction in sodium chloride intake can prevent hypertension, but it is reverse in case of potassium intake [[31], [32], [33]]. In the present study, four important electrolytes, sodium, potassium, chloride and bicarbonates were measured. Although, 97% participants reported for taking moderate salt in their diet, however, the test medicine did not alter any electrolytes balance.

In the present study, a thorough hematological, biochemical, electrophysiological and radiological investigations were done in baseline and after 8 week of treatment with Heart Revival. The QT interval on the surface ECG signifies the addition of action potential of ventricular myocytes. The QT-interval prolonging drugs should not be recommend in patients with pre-existing CVDs, particularly ventricular arrhythmias or with metabolic abnormalities [34]. In the present study, 8 week ingestion of trial medicine did not altered QT intervals of trial participants that clearly indicate that Heart Revival is devoid from any cardio-toxicity. Therefore, the outcome results point out that Heart Revival did not has any myelosuppressive effect or hematopoietic actions in hypertensive patients. Furthermore, it did not alter/modify liver and renal metabolisms or excretory functions.

From the above findings it is therefore speculated that 8 active components of Heart Revival acts on blood vessels, heart, hepatic and renal tissues concurrently to facilitate vasodilatation, to supply more blood and oxygen into myocardium, to prevent the access of calcium into heart muscles and arteries, to inhibit the function of HMG-CoA reductase in hepatocytes, to regulate excess ions through renal function.

A limitation of this trial is that we were unable to include the patients with severe hypertensive and CVDs due to safety grounds as the interventive medicine had no previous clinical data to support it; this is a standard limitation of clinical trials.

## 5 Conclusion

Heart Revival a polyherbal formulation significantly lowered blood pressure and lipids profile in hypertensive and hypercholesterolemic patients with in 8-weks. Moreover, it also drastically and effectively minimized fatigue, chest discomfort and breathing trouble in hypertensive patients. Furthermore, it is harmless for hypertensive patients. Therefore, Heart Revival is a safe and effective medicine for hypertension and hypercholesterolemia and may be helpful in the management CVDs. Further investigation is recommended in larger population with more variables to strengthen the present findings.

## Funding sources

This research work was financially supported by M/s Health Reactive, Jaipur, Rajasthan, India.

Conflict of interest

None.

Declaration of generative AI in scientific writing

The authors declare that no artificial intelligence (AI) techniques or technologies were utilized throughout the writing process or to enhance the language and comprehension of this paper.

## Author contribution

(i) Conception and design of study-S.P., T.K.B., U.J., T.K.S.; (ii) Acquisition of data- S.P., T.K.B., S.S., S.B.; (iii) Analysis and interpretation of data- S.P., T.K.B, U.J., T.K.S.; (iv) Drafting the manuscript- T.K.S.; (v) Review and finalized the manuscript- S.P., T.K.B.,S.B., U.J., T.K.S.
